# Constructing a Theoretical Hybrid Care Pathway From Stakeholder Perspectives: User-Centered Design Study

**DOI:** 10.2196/93735

**Published:** 2026-10-30

**Authors:** Ruben Knevels, Jelle Schils, Sarah Haesevoets, Julie Vranken, Chris Burtin, David Ruttens, Femke Wouters

**Affiliations:** 1 Faculty of Medicine & Life Sciences Hasselt University Diepenbeek, Flanders Belgium; 2 Limburg Clinical Research Center / Mobile Health Unit Hasselt University Diepenbeek, Flanders Belgium; 3 Department Future Health Ziekenhuis Oost-Limburg Genk, Flanders Belgium; 4 REVAL - Rehabilitation research center Faculty of Rehabilitation Sciences Hasselt University Diepenbeek, Flanders Belgium; 5 Department of Respiratory Medicine Ziekenhuis Oost-Limburg Genk, Flanders Belgium

**Keywords:** care pathway, chronic obstructive pulmonary disease, COPD, digital health, hybrid care, remote patient monitoring

## Abstract

**Background:**

Chronic obstructive pulmonary disease (COPD) is characterized by acute exacerbations that frequently result in hospitalization, highlighting the need for improved follow-up care. Hybrid care incorporates digital health strategies such as remote patient monitoring (RPM). This could offer the opportunity to detect early signs of disease worsening by remotely tracking selected health indicators. However, it is unknown how RPM can effectively be integrated into current practice.

**Objective:**

This study aims to cocreate a theoretical hybrid COPD care pathway integrating RPM into the existing care trajectory, considering the expertise of participating care pathway stakeholders.

**Methods:**

This 2-phase study was conducted between May and August 2025. At first, semistructured interviews were conducted with stakeholders (ie, patients and health care professionals [HCPs]) involved in COPD care to map the existing care pathway in a single tertiary care hospital in Belgium. Patients were approached during hospitalization based on predefined eligibility criteria, whereas HCPs were recruited through convenience sampling. In phase 2, patient representatives and HCPs were recruited through patient-organization networks and professional referral, respectively, using convenience sampling, and participated in separate interactive workshops. These workshops reviewed existing care pathway phases and HCP roles and explored the integration of RPM, including timing, duration, implementation, data monitoring, and follow-up responsibilities, to codevelop a theoretical hybrid care pathway. Interview and workshop data were independently reviewed by 2 researchers to extract information on care pathway elements, stakeholder roles, limitations, and proposed adaptations. Findings were organized according to the relevant care pathway phase, independently summarized and compared, with discrepancies resolved through discussion and consensus. Interview findings informed the development of an initial infographic representing the existing care pathway, which was subsequently adapted based on workshop findings to represent the proposed cocreated theoretical hybrid care pathway.

**Results:**

In total, 10 stakeholders participated in the interviews (5 patients and 5 HCPs), and 6 stakeholders participated in the workshops (3 patient representatives and 3 HCPs). The existing COPD care pathway involves multiple stakeholders whose activities can be organized into 5 functional domains: support, treatment, medical advice, patient education, and monitoring. Stakeholders can be divided into hospital-based, community-based, or cross-setting. The workshop participants identified the need for a stakeholder dedicated to RPM activities in order to integrate RPM strategies into the care pathway. This individual could be responsible for all RPM-related activities (ie, RPM device installation, providing RPM-related education, data review, alert management, and RPM discontinuation) and could serve as the bridge between patients and physicians, ensuring close communication.

**Conclusions:**

This study resulted in a cocreated theoretical hybrid COPD care pathway. Stakeholders highlighted the potential value of a dedicated RPM role to coordinate and deliver RPM activities as part of the COPD care pathway. Further research is needed to assess its feasibility and clinical applicability.

## Introduction

Chronic obstructive pulmonary disease (COPD) is a progressive respiratory condition characterized by recurrent episodes of acute symptom worsening, known as acute exacerbations of COPD (AECOPD) [1]. These are defined as “an event in the natural course of the disease characterized by a change in the patient’s baseline dyspnea, cough, and/or sputum that is beyond normal day-to-day variations, is acute in onset, and may warrant a change in regular medication in a patient with underlying COPD” [1]. While approximately 18% to 33% of patients with COPD Global Initiative for Chronic Obstructive Lung Disease stage 3 or 4 are hospitalized at least once per year, rehospitalizations represent an even greater challenge [2]. A large cohort study of 15,191 patients with AECOPD reported that 35.1% of patients hospitalized for AECOPD were readmitted within 90 days [3]. AECOPD is not only a leading cause of costly emergency room (ER) visits and hospitalizations but is also associated with poor quality of life [4].

Effective management of COPD, especially during and after AECOPD events, requires coordination across multiple care settings, patient engagement, and timely intervention. Care pathways represent a potential organizational strategy for achieving these aims [5]. A care pathway can be defined as a complex intervention for the mutual decision-making and organization of a care process for a well-defined group of patients during a well-defined period [5]. Their use offers the advantages of standardizing best practice, streamlining care processes, and providing a structural framework for collaboration between care pathway stakeholders [6]. Additionally, in COPD, care pathways have shown the potential to reduce complications, readmissions, and length of stay [7]. Given that no globally established guidelines exist for structured COPD care, clinical practice differs between centers and countries [8].

Hybrid care, which combines traditional in-person care with digital health modalities such as remote patient monitoring (RPM), is being increasingly adopted across various health care domains. Evidence suggests that hybrid approaches can enhance access, patient engagement, and continuity of care, particularly in the management of chronic diseases [9-11]. In addition, they may offer a scalable solution to reduce ER visits and exacerbation-related readmissions in patients with COPD [12]. Despite the growing adoption of hybrid care models, evidence on how to effectively integrate hybrid care components into existing care pathways remains limited. Furthermore, it is unclear how these pathways should be adapted to ensure that quality, safety, and coordination are maintained [11].

The aim of this descriptive co-design study was to (1) map the current COPD care pathway, including stakeholder role delineation and perceived bottlenecks, and (2) cocreate a theoretical hybrid care pathway for patients with COPD after an AECOPD event, incorporating digital health strategies based on stakeholder perspectives.

## Methods

### Study Design: 2-Phase Design

A 2-phase descriptive co-design approach was used to develop a theoretical hybrid COPD care pathway incorporating digital health strategies. Semistructured interviews and interactive workshops were conducted with patients with COPD, patient representatives of COPD, and health care professionals (HCPs) involved in COPD care at a tertiary care center and its regional care network in Belgium. The study specifically targeted people who experience COPD exacerbations, defined as individuals experiencing AECOPD who required hospitalization.

Data collection occurred in consecutive phases. Recruitment continued until sufficient information had been obtained to support the mapping of the care pathway. Information sufficiency was assessed at the level of the overall care pathway rather than within each individual stakeholder group. Recruitment continued until the interviews consistently confirmed previously identified care pathway elements and did not substantially contribute new information relevant to the study’s focus. For stakeholder groups represented by multiple participants, consistency of information across interviews was considered when assessing whether additional interviews were likely to contribute new pathway elements. Groups represented by a single individual were considered specific informants because of their distinct roles and expertise in COPD care or hybrid care. In these interviews, information sufficiency could therefore not be assessed through within-group repetition. Instead, their contributions were assessed in relation to the broader pathway information obtained from other participants and their specific relevance to the pathway components with which they were involved.

Both interviews and workshops were conducted by a postdoctoral researcher (FW) with a PhD in Biomedical Sciences, accompanied by a second researcher (RK), who took field notes and digitally recorded the sessions. Both researchers had a background in digital health research. They had no direct clinical care relationship with the participants. Sessions were audio-recorded with participant consent, and recordings were destroyed following manual, clean verbatim transcription. Participant checking was not performed.

In the first phase, semistructured interviews were conducted to gather the perspectives of both patients and care pathway stakeholders on the existing COPD care pathway. Insights from these interviews informed the visual representation of the care pathway in the form of an infographic. In the second phase, this infographic was used as a guide during the workshops. A new theoretical hybrid care pathway was developed, informed by stakeholder expertise. This new hybrid care pathway was again visualized in the form of an infographic (Figure 1).

**Figure 1:**
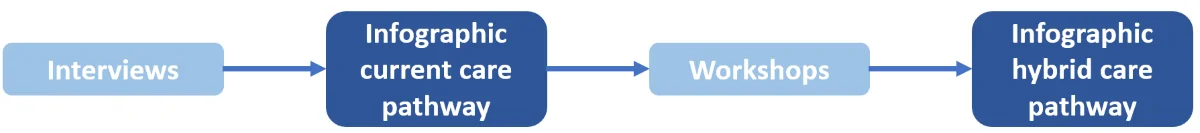
Study design.

This study adhered to the COREQ (Consolidated Criteria for Reporting Qualitative Research) guidelines [13]. The COREQ checklist is provided in Multimedia Appendix 1.

### Phase 1: Interviews

#### Recruitment

Eligible interview participants included patients hospitalized due to AECOPD and HCPs involved in COPD management or RPM. Patient selection was based on predefined inclusion and exclusion criteria. All patients had a confirmed diagnosis of COPD by spirometry (Tiffeneau index <0.7) and a history of multiple AECOPD events, including hospitalization due to AECOPD within the preceding year. Patients hospitalized due to AECOPD at Ziekenhuis Oost-Limburg were screened for eligibility by DR. Eligible patients were approached in order of hospital admission. HCPs were eligible if they were actively involved in the care of patients with COPD and had at least 2 years of experience in COPD care. HCPs were identified based on their active involvement in COPD care and selected to represent different professional roles in the COPD care pathway. Potential participants were identified through professional contacts within the clinical networks and contacted by email with an invitation to participate. Recruitment therefore followed a convenience sampling approach based on the accessibility and willingness of eligible HCPs to participate. Eligible HCPs included all professionals actively involved in the care of patients with COPD (ie, pulmonologists, physiotherapists, occupational therapists, and a dedicated COPD nurse) and did not have to be associated with Ziekenhuis Oost-Limburg. To support the development of a hybrid care pathway, an additional interview was conducted with a telemonitoring nurse from Ziekenhuis Oost-Limburg who had extensive experience in implementing hybrid care in the Cardiology department.

#### Interview Procedures

Semistructured, face-to-face, one-on-one interviews were conducted to explore the perceptions of patients and HCPs regarding the current COPD care pathway. All interviews were carried out by the same researcher to ensure consistency. Interview guides were developed by FW by first identifying the information required from each participant group to map the existing COPD care pathway. Questions were then formulated to address the relevant experiences, roles, and perspectives of each group. Separate guides were developed for patients, physicians, occupational therapists and physiotherapists, the COPD nurse, and the RPM nurse. The guides consisted of open-ended questions covering relevant aspects of COPD care and were tailored to the specific roles and experiences of each participant group. Because the study involved RPM using digital technology, additional questions were asked to evaluate participants’ digital literacy. The interview guides for patients, physicians, occupational therapists and physiotherapists, COPD nurses, and RPM nurses are provided in Multimedia Appendices 2-6, respectively.

Prior to each interview, patients were informed that the purpose of the study was to explore their experiences and perceptions of the care they received following previous hospitalizations. For HCPs, the goal was to gain insights into their professional roles and perspectives on the COPD care pathway. Interviews used open-ended questions organized into categories, including postdischarge treatment, follow-up, perceived bottlenecks, symptoms, involvement of informal caregivers, and potential areas for improvement in care. All interviews were conducted at the tertiary care center Ziekenhuis Oost-Limburg in Genk, Belgium.

#### Data Analysis

Interview transcripts and accompanying field notes were reviewed independently by FW and RK. Each researcher reviewed the transcripts and notes to identify information relevant to the existing COPD care pathway at Ziekenhuis Oost-Limburg, focusing on (1) the different phases of care, (2) the stakeholders involved in each phase, (3) the actions and responsibilities of each stakeholder, (4) perceived limitations or bottlenecks in the current care pathway, and (5) suggestions for improving current care processes. Formal qualitative coding was not performed; instead, relevant information was systematically extracted and organized according to the corresponding phase of the care pathway. Each researcher subsequently developed an independent summary of the identified care pathway elements and stakeholder perspectives. These summaries were compared to identify areas of agreement and discrepancy. Discrepancies were discussed by FW and RK until consensus was reached. The resulting synthesis was used to construct the initial infographic representing the existing COPD care pathway, including different phases, involved stakeholders, and their respective actions and responsibilities. Illustrative quotes were selected to reflect the identified perspectives and provide examples of the findings.

### Phase 2: Workshops

#### Recruitment

Eligible workshop participants included Dutch-speaking representatives of patients with COPD, informal caregivers of patients with COPD, and HCPs. Patient representatives were selected over hospitalized patients to provide perspectives informed by broader experiences with COPD and health care services, rather than experiences based solely on individual clinical status or hospitalization history. In addition, through their ongoing activities and interactions with other people living with COPD, they are exposed to a variety of patient experiences and perspectives beyond their own. Patient representatives were members of COPD vzw, a Flemish organization representing patients with COPD. Representatives were contacted by email and invited to participate in the workshop. Workshop HCPs were eligible if they were actively involved in the care of patients with COPD and had at least 2 years of experience in COPD care. HCPs were identified based on their active involvement in COPD care and selected to represent different professional roles in the COPD care pathway. Participants were invited by email and recruited using a convenience sampling approach. To maximize data richness and encourage honesty about challenges, separate workshops were organized for patient-side stakeholders and HCPs.

#### Workshop Procedures

Each workshop was an interactive, semistructured session designed to engage participants in collaboratively considering the implementation of digital health solutions within the current COPD care pathway. After the study objectives were introduced, participants reviewed and verified care pathway phases and HCP roles. Next, participants discussed the infographic representing the current care pathway and explored transitions toward a hybrid model. In this hybrid model, RPM was introduced by the researchers as a nonintrusive wearable device (eg, a smartwatch) capable of measuring multiple vital parameters. Specific devices, parameters, and alert protocols were not further explored. Discussion topics included role delineation, stakeholder responsibilities, the period of remote monitoring, the integration of remote monitoring into routine COPD care, and potential hurdles to implementation. The workshop guides for HCPs and patient representatives are provided in Multimedia Appendix 7.

#### Data Analysis

Workshop transcripts and notes were independently reviewed by FW and RK using a descriptive approach similar to that used with the interviews. The analysis focused on proposed changes to the existing care pathway and the integration of RPM and other digital health strategies, including (1) changes in stakeholder roles and responsibilities, (2) communication and follow-up processes, (3) monitoring activities, and (4) points of interaction between stakeholders. Formal qualitative coding was not performed; instead, relevant information was systematically extracted and organized according to the corresponding phase of the care pathway. FW and RK independently developed descriptive summaries of the proposed adaptations and compared their findings. Discrepancies were discussed and resolved through consensus. The agreed findings were then mapped onto the initial infographic to identify which components of the existing pathway should be retained, modified, added, or removed. This iterative process resulted in a second infographic representing the proposed theoretical hybrid COPD care pathway. Illustrative quotes were selected to reflect the identified perspectives and provide examples of the findings.

### Statistical Analysis

Data processing and statistical analysis were performed using R (version 4.6.0; R Foundation for Statistical Computing, Vienna, Austria). Descriptive statistics are presented as mean (SD) or number (%), as appropriate.

### Ethical Considerations

The study protocol complied with the principles of the Declaration of Helsinki and was approved by the medical ethics committee (ie, Ziekenhuis Oost-Limburg, Genk, Belgium; B3712025000001). Written informed consent was obtained from all participants prior to data collection.

## Results

### Participant Characteristics

Participants were recruited between May and August 2025. A total of 5 patients with COPD and 5 care pathway stakeholders were approached for the interviews. As no participants declined the invitation, 5 patients with COPD and 5 care pathway stakeholders participated in the interviews (Table 1). The interviews with patients lasted 11.8 (range 8.0-32.3) minutes, and those with stakeholders lasted 23.2 (range 15.3-45.3) minutes. For the workshops, 3 patient representatives and 3 HCPs were invited. No participants declined the invitation. Workshops with patient representatives lasted 1 hour 38 minutes, and workshops with HCPs lasted 1 hour 28 minutes. One patient representative was also active as an informal caregiver. None of the interviewees took part in the workshops. All HCPs reported daily internet use, and 4 out of 5 reported daily smartphone use, compared with 2 out of 5 and 1 out of 5 patients, respectively. Only 1 HCP received data from wearables or remote monitoring, whereas none of the patients did.

**Table 1:** Characteristics of the interview and workshop participants.

Characteristic	Interview		Workshop	
	Patient (n=5)	HCP^a^ (n=5)	Patient representative (n=3)	HCP (n=3)
Age (years), mean (SD)	65.0 (4.1)	38.6 (14.5)	71.7 (3.1)	53.3 (14.2)
Male, n (%)	3 (60)	2 (40)	2 (66.7)	2 (66.7)
Experience^b^ (years), mean (SD)	N/A^c^	6.7 (6.2)	10.7 (8.1)	24.0 (4.9)
Time since diagnosis (years), mean (SD)	7.6 (4.3)	N/A	N/A	N/A

^a^HCP: health care professional.

^b^Number of years of experience is calculated as the number of years a patient representative has participated as a representative of patients with COPD or an HCP has worked in COPD-related care.

^c^N/A: not applicable.

### Care Path Overview

HCP participants indicated that the current care pathway for people who experience COPD exacerbations can be set out in general terms. However, they emphasized that individual adjustments are necessary depending on patients’ medical history, pulmonary function test results, and individual illness experience.

Overall, the interviews indicated that patients may either have been formally diagnosed with COPD before their first AECOPD event or encounter the disease for the first time in the context of an AECOPD hospitalization. While mild AECOPD events can often be managed in primary care, severe cases often require hospital admission. After a period of hospitalization, the patient stabilizes and is discharged from the hospital. In the care pathway described by participants, the discharge decision is based on a clinical evaluation that includes infectious parameters, general blood work, and the need for supplemental oxygen. If necessary, the patient may be discharged with temporary supplemental oxygen at home. However, this clinically determined point of discharge did not always correspond to patients’ perceived readiness to return home. Several patients described feeling insufficiently prepared for discharge, highlighting a gap between clinical stability and perceived readiness for home recovery (quote 1 in Textbox 1).

**Illustrative quotes from interviews and workshops with patients, patient representatives, and health care professionals.**

**Quote 1: patient 4 (interview)**

- For me, it (being discharged) was mentally very challenging. I continually questioned myself whether I was ready for it.

**Quote 2: patient representative (workshop)**

- The pharmacist is actually someone who is just as close to the patient as the GP. That’s where you go for your medication, and that’s where your partner goes for their medication.

**Quote 3: health care professional (workshop)**

- Wherever a home care nurse visits, education can be provided, but we still miss a group of patients we never reach. All patients, however, visit the pharmacist. Not all physicians know that they must write a prescription for inhalation-therapy education. The pharmacist needs that prescription to be compensated.

**Quote 4: home nurse (workshop)**

- The required level of monitoring vital signs should be determined on an individual basis. However, a challenge is that many patients do not receive home nursing care, particularly when no technical nursing or hygiene-related support is required. In these cases, home nurses are often not involved. This creates a gap regarding patient education and support, as these activities are not reimbursed as home nursing services. From the patient’s perspective, charging €10 per day for this may represent a considerable financial burden, even though a daily cost ceiling applies. At the same time, this fee would not cover the involvement of a home nurse. This creates a challenge, despite the fact that such support could be highly valuable for patient follow-up.

**Quote 5: patient representative (workshop)**

- When you leave the hospital, it feels like something hits you on the head. You only remember that you won’t be able to manage for a while, and everything else is gone. If someone else is with you, they can put things into perspective and remember the details. But as a patient, you’re told so much that you simply can’t take it all in anymore.

**Quote 6: pulmonologist (interview)**

- In case of disease worsening, we always advise patients to see a physician. In most cases, this is their GP, but long waiting times can allow their condition to worsen. Having a way to monitor patients remotely, without adding excessive medical burden or cost, could be crucial.

Besides feeling unprepared, patients reported experiencing a lack of information, guidance, and follow-up after hospitalization for AECOPD. In the local care setting, discharge letters typically contain the necessary clinical details and are provided to patients or transmitted electronically to their general practitioner (GP). However, patients reported that the information they received was largely limited to their current pharmacological regimen. Moreover, they noted a lack of guidance regarding potential postdischarge symptoms, appropriate self-management strategies, and clear instructions on whom to contact in the event of clinical deterioration.

Although the exact point at which recovery begins is not always clear, 2 possible scenarios were identified during this phase. In the first, the disease suddenly worsens, often resulting in a new visit to a GP or ER, with potential hospitalization. In the second scenario, recovery progresses into a stable phase, during which patients should receive at least 1 annual outpatient follow-up that includes a pulmonology consultation and pulmonary function testing. Depending on individual needs, certain components of the care pathway may be added or omitted (Figure 2).

**Figure 2:**
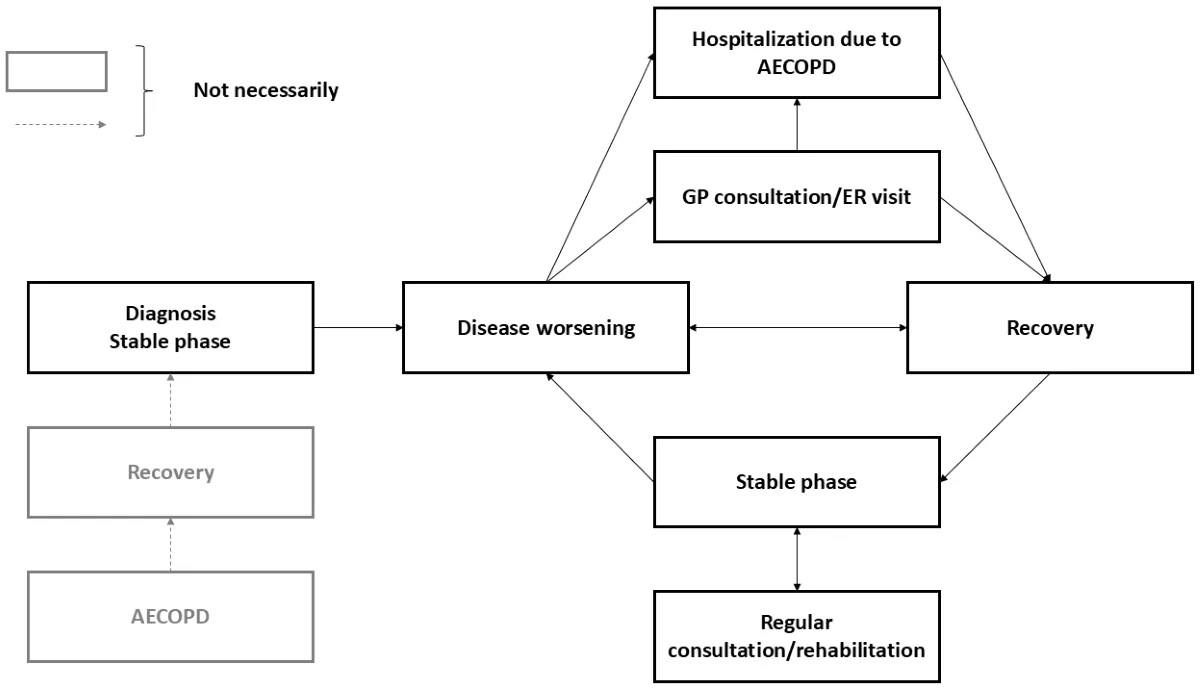
General clinical trajectory of a patient experiencing an acute exacerbation of chronic obstructive pulmonary disease (AECOPD). GP: general practitioner; ER: emergency room.

### Care Pathway Stakeholders

The care pathway for patients who frequently experience COPD exacerbations can be represented by the vicious circle in the general clinical COPD trajectory (Figure 2). Across this pathway, 10 key stakeholders were identified, which can be categorized into 3 groups based on their primary setting of interaction: hospital-based, community-based, and cross-setting (Multimedia Appendix 8). The stakeholder roles described reflect responsibilities, activities, and interactions reported by study participants in relation to the existing COPD care pathway. These descriptions therefore reflect participants’ experiences and perceptions of how local care was organized in practice and are not intended to define the formal scope of practice of each profession.

### Hospital-Based Stakeholders

The pulmonologist is primarily responsible for patient treatment, including prescribing medication and interpreting spirometry results. Additionally, participants reported that the pulmonologist plays a critical role in patient education, discussing lifestyle modifications and pulmonary rehabilitation. While most hospital-based interactions occur during periods of hospitalization, annual ambulatory follow-ups were reported to be recommended to assess medication efficacy and lung function progression.

The hospital nurse was mentioned as contributing both during hospitalization and in ambulatory follow-up. During hospitalization, the hospital nurse provides direct patient care and education. During outpatient visits, participants described the primary task of the nurse as performing spirometry testing.

The dietitian was mentioned as providing dietary counseling, particularly for patients experiencing unintentional weight loss or gain, thereby supporting nutritional optimization as part of COPD management.

According to interviewed participants, the social worker evaluates the patient’s living environment, supports discharge planning, and offers psychosocial support when needed.

### Cross-Setting Stakeholders

Although their role may appear self-evident, pharmacists were recognized by both patients and HCPs as essential contributors to the COPD care pathway. Beyond supplying prescribed therapies, pharmacists were reported to be well-positioned to guide patients and informal caregivers in the correct use of inhalers and other respiratory treatments. Their frequent and accessible contact with patients allows them to reinforce key aspects of disease management and support adherence to therapy (quote 2 in Textbox 1). Notably, a participating HCP mentioned that, in Belgium, these educational services may be eligible for reimbursement when appropriately prescribed by the patient’s treating physician (quote 3 in Textbox 1).

Another important stakeholder who can be present in both hospital-based and community-based settings is the physiotherapist. In the hospital, the role of the physiotherapist was described as focusing on patient mobilization, physiotherapy-related education, inhaler use, and general disease management. After hospital discharge, according to participants, the physiotherapist is responsible for guiding the patient through pulmonary rehabilitation.

The physiotherapist primarily focuses on restoring mobility, physical performance, and overall body function. The occupational therapist, in contrast, was described by participants as supporting patients in regaining or maintaining independence in activities of daily living (ADL). In addition to ADL training, participants mentioned that the occupational therapist may provide education on energy preservation, breathing techniques, and relaxation methods. For patients at increased risk of falls, targeted preventive interventions are also implemented.

### Community-Based Stakeholders

During daily life, ongoing medical management was described by participants as being primarily coordinated by the GP. As the patient’s primary point of access to health care, the GP was described as providing comprehensive and ongoing care, addressing both acute issues and long-term conditions. Participants indicated that the GP’s role includes discussing lifestyle modifications, educating patients on medication use and symptom monitoring, and supporting overall disease control. Participants also highlighted that, when patients experience worsening respiratory symptoms, Belgian local practice allows the GP to perform diagnostic tests, such as a C-reactive protein test, to help identify potential infectious exacerbations.

Alongside the GP, the home care nurse was described by participants as playing a key role in supporting patients in their own homes. Within the care pathway described by participants, home care nurses were reported to provide professional nursing care, play a role in therapy- and lifestyle-related education, and assist with symptom management and overall disease management. Participants also indicated that home care nurses may monitor vital signs and other clinical indicators, enabling early detection of potential deterioration and timely intervention. However, participants noted that their involvement is generally limited to situations in which patients present with additional care needs, most commonly due to comorbid conditions that require home care nursing services (quote 4 in Textbox 1).

The final stakeholder in the COPD care pathway is the informal caregiver. Although participants noted that not all patients have access to unpaid, nonprofessional support, multiple stakeholders recognized the important role informal caregivers often play in daily life management. Participants described their responsibilities as varying according to the patient’s needs and the nature of their relationship, potentially including personal care, emotional support, assistance with household tasks, and monitoring of the patient’s health status. Study participants reported that the contributions of informal caregivers are often underrecognized by health care professionals. In particular, they highlighted that patients may feel overwhelmed during hospital visits and struggle to retain important information. For instance, participants described the presence of an informal caregiver or family member during the initial diagnosis, often perceived as life-altering, as helping patients better understand and remember key instructions and explanations provided by the pulmonologist (quote 5 in Textbox 1).

While most stakeholders are continuously present, certain stakeholder roles were mentioned as being embedded only as needed, such as those of the dietitian, social worker, home care nurse, and informal caregiver. Although these stakeholders play valuable roles, their involvement depends on the patient’s personal circumstances, preferences, and individual care needs.

### Limitations of the Current Care Pathway

Stakeholders identified several organizational and communication challenges within the current COPD pathway. These included the insufficient or time-limited involvement of physiotherapists and occupational therapists during and after hospitalization, inadequate patient and informal caregiver education, and a lack of structured follow-up at home, commonly referred to as the “black box” phase of postdischarge care. Both patients and professionals emphasized that education provided during acute hospitalization is often forgotten due to emotional strain and should therefore be repeated and reinforced during follow-up visits. In addition, physicians noted that referrals to physiotherapists and dietitians are not based on clearly defined criteria; instead, they largely depend on clinical judgment and physician preference. As a result, physiotherapy, particularly outpatient physiotherapy, is often initiated too late, even though earlier referral and intervention might be beneficial for patient outcomes. Moreover, communication between primary and secondary care has several limitations. Information is often lost or repeatedly requested, and relevant clinical details are not consistently transferred across care settings because different electronic health systems are unable to exchange data effectively. On the other hand, disease deterioration is often accompanied by patient anxiety and hesitation to seek help, sometimes reinforced by a perceived sense of failure when symptoms cannot be managed independently. This uncertainty may delay timely action, underscoring the need for objective RPM to support patients in recognizing clinically significant changes and responding appropriately (quote 6 in Textbox 1).

### Development of a Hybrid COPD Care Pathway

This new theoretical hybrid care pathway focuses on patients who experience hospitalization due to AECOPD, as they place the greatest burden on the COPD care system. Stakeholders across primary and secondary care agreed that RPM strategies should be initiated and coordinated within secondary care, where clinical expertise and data interpretation capacity are concentrated. Follow-up was considered most effective when led by a dedicated hospital-based nurse (eg, a COPD coach), who serves as the primary individual responsible for all RPM-related activities (quote 7 in Textbox 2). In addition, this individual could act as the first point of contact for patients experiencing worsening COPD-related symptoms, including those who are not enrolled in an RPM program (quote 8 in Textbox 3). RPM-related activities include installing the RPM devices, providing RPM-related education, reviewing RPM data, managing alerts, and discontinuing RPM when appropriate. While the COPD coach handles the practical aspects, close collaboration with the treating physician and regular communication with the GP remain essential. In addition to RPM management, the COPD coach can also play a key role in enhancing disease- and medication-related education and serve as a key contact person for both patients and their informal caregivers (Multimedia Appendix 8).

**Quote 7: health care professional (workshop).**

**Interviewer:** Who should be responsible for reviewing the RPM-generated alerts?

**Pulmonologist:** Initially, this would be a nurse, who then reports back to the physicians (pulmonologists).

**General practitioner:** In our setting as well, especially if multiple conditions were being monitored, it would be a practice nurse who would perform the initial triage of alerts.

**Home nurse:** With telemonitoring, we are currently considering having an on-call nurse review the data once per day and escalate issues as needed.

**Pulmonologist:** I think it would be easier for our (pulmonology) department to assign someone to this task rather than shifting it to you in primary care.

**General practitioner:** Yes, we cannot hire someone for every pathology.

**Pulmonologist:** We want to reduce your workload. That already starts with a COPD exacerbation, so inclusion in RPM happens in the hospital. Therefore, it seems logical for this responsibility to remain with us (the hospital).

**Quote 8: health care professional (workshop).**

**Interviewer:** Who should be the first point of contact for the patient?

**Pulmonologist:** A study nurse or a reference nurse, like we have in our oncology department (onco-coach). In case the patient experiences any issues, the patients contacts the nurse, who tries to solve the problem or contacts us in case of a medical issue.

Workshop participants emphasized that RPM should be considered a complementary tool rather than a replacement for direct patient contact. Although physiological parameters provide valuable insights, they do not fully capture the patient’s clinical condition. Participants highlighted the importance of maintaining telephone or face-to-face interactions, as communication allows HCPs to assess additional aspects, such as the patient’s breathing, speech, and overall presentation, which are essential for interpreting the clinical relevance of monitored parameters (quote 9 in Textbox 4).

**Quote 9: health care professional (workshop).**

**Pulmonologist:** Is telephone consultation included somewhere in the infographic? We receive many phone calls from patients, and based on these conversations, we assess whether a patient should come in for a consultation or whether, for example, a short course of medication is appropriate if we know the patient well.

**Interviewer:** Could this be replaced by increased RPM?

**Pulmonologist:** No, home monitoring would complement this or provide an initial indication, but when you speak to a patient by phone, you also hear many relevant aspects: how they talk, how they breathe, and they can explain their situation.

**Home nurse:** Parameters are important, but they do not tell the whole story. With telemonitoring, when a parameter suggests that something may be wrong, it remains important to see or hear the patient directly to assess whether that value is clinically meaningful. RPM can serve as a supportive tool.

Primary care providers, particularly home care nurses, were viewed as having a supportive role, assisting with device troubleshooting or connectivity issues and communicating with the hospital nurse when necessary. As not all patients receive home care nursing, direct access to the COPD coach was considered essential.

GPs preferred to remain informed of their patients’ participation in an RPM program but did not wish to interpret continuous data streams. Instead, they favored receiving periodic summary reports, with stakeholders specifically suggesting a one-page summary (quote 10 in Textbox 5) offering an overview of the patient’s clinical status. HCP workshop participants reached a consensus that, within the proposed pathway, RPM data and alerts would therefore be reviewed and triaged by the designated in-hospital RPM team, with clinically relevant findings escalated to the appropriate pulmonologist (quote 7 in Textbox 2). This approach facilitates information sharing and continuity of care.

**Quote 10: health care professional (workshop).**

I think those data should be available when the patient sits in front of you during a consultation, so you can integrate the data with everything else. When you’re with the patient, you want to see it: that little graph of the oxygen saturation or breathing rate. There should already be an A4 sheet ready with those data on it.

Currently, RPM is not embedded in the daily management of patients with COPD at our care center, but it is likely to be more beneficial for those with severe disease or multiple comorbidities. However, participants indicated that patients without multiple comorbidities could also benefit from closer monitoring, representing a significant opportunity for improved care. Educating patients to enable self-monitoring is essential, and monthly follow-up for more stable patients would be desirable to ensure continuity and early detection of potential issues.

The hybrid COPD care pathway is presented as an infographic (Multimedia Appendix 8). It is organized into the main phases of the care pathway (ie, hospitalization, recovery, stable phase, and disease worsening), mirroring the circular trajectory shown in Figure 2. For each phase, the infographic summarizes the key activities of all stakeholders, grouped into 5 functional domains: support, treatment, medical advice, patient education, and monitoring. Changes from the current care pathway to the hybrid model are indicated with an asterisk.

### Potential Barriers to RPM Implementation

Several potential barriers to RPM implementation were identified during the workshops, including low digital literacy, limited disease awareness, and affordability. Low digital literacy was highlighted as an important consideration in this population (quote 11 in Textbox 6). However, participants discussed that RPM could remain feasible if the system were designed to be as simple and user-friendly as possible, requiring minimal effort from patients. Limited disease awareness was also identified as a potential challenge, as some patients may be reluctant to engage in monitoring or to be continuously confronted with their disease status (quote 12 in Textbox 7). Furthermore, participants emphasized that alerts generated by the RPM system should be minimized and, where possible, should not be directly visible to or burdensome for patients.

**Quote 11: health care professional (workshop).**

**Interviewer:** What are potential barriers for patients to use RPM?

**Home nurse:** I think the main challenge is digital illiteracy.

**Pulmonologist:** Yes, but perhaps patients should not have to do much themselves?

**General practitioner:** If it is a watch that performs many tasks automatically, this should not cause many problems.

**Quote 12: health care professional (workshop).**

**Home nurse:** Another potential barrier is disease awareness. Patients may be reluctant to be monitored in this way. Similar to what we currently observe, some patients do not seek care from their general practitioner or hospital because they do not want to know how severe their disease is. RPM may confront them with their condition more directly.

**Interviewer:** So, they may feel that they are being confronted too much with the reality of their disease?

**General practitioner and home nurse:** Yes, indeed.

## Discussion

### Principal Findings

This study mapped the current COPD care pathway, delineated stakeholder roles, and developed a stakeholder-informed theoretical hybrid COPD care pathway incorporating RPM, while identifying key considerations for its integration into routine COPD care. Stakeholders were defined as individuals involved in the organization, delivery, or governance of COPD care. While internationally established guidelines exist for the management of COPD, there is no globally standardized COPD care pathway. Nevertheless, these guidelines recommend common principles that should underpin COPD care, including appropriate pharmacological treatment, smoking cessation, and annual vaccination. In addition, individualized assessment of nutritional status and mobility, encouragement of pulmonary rehabilitation, and evaluation of the need for home supplemental oxygen therapy should be performed [1]. However, the organization and implementation of these principles within care pathways often rely on the clinical judgment of physicians or locally developed internal hospital guidelines [8]. Consequently, substantial variation may occur between patient trajectories, depending on the country, available resources, and health system organization [8].

Although there are often no clear guidelines for COPD trajectories, evidence suggests that structured care pathways can improve care and outcomes for patients, as well as reduce unnecessary resource use and associated costs for health care providers [14,15]. The proposed theoretical hybrid care pathway is based on the COPD care pathway at a tertiary care hospital with an academic character (ie, Ziekenhuis Oost-Limburg, Genk) in Belgium and may differ from practices in other clinical institutions. Nevertheless, many of the general insights obtained remain relevant. In particular, the overarching responsibilities and the role of the COPD coach within the proposed theoretical hybrid care pathway may provide a useful starting point for consideration in other care settings and may inform the future development of similar care pathways. However, the specific delineation of this role, including task allocation, integration within existing care teams, and organizational arrangements, is highly context- and institution-specific. As these aspects were beyond the scope of the workshops, they should be further defined and adapted by individual health care institutions during local implementation.

In the development of the hybrid care pathway, the researchers specified that RPM would be performed using a wearable device capable of measuring multiple vital parameters, without further specifications. The proposed hybrid care pathway should therefore be understood as an organizational framework for integrating RPM into COPD care rather than a device-specific protocol. It does not prescribe or evaluate specific devices, monitored parameters, alert thresholds, or escalation protocols, which should be defined and evaluated in subsequent research. Given that various hybrid RPM approaches have been evaluated in the literature, with varying results, further research should specify the components and structure of RPM in COPD [16]. Nevertheless, the integration of RPM into chronic care pathways is not a mere technical add-on but necessitates a reconfiguration of roles, responsibilities, and interactions within the care team. Previous literature on real-world implementations showed that it requires a fundamental reorganization of clinical workflows, staffing, and coordination structures [17,18]. For instance, in a multicenter European oncology program covering 33 cancer centers in France and Belgium, a remote symptom-monitoring pathway based on weekly patient-reported outcomes was successfully deployed after explicit roles were defined, alert-management workflows were established, and the RPM process was embedded into routine care using implementation science methods [19]. Similarly, in chronic heart failure care, surveys found that more than 80% of cardiology departments created dedicated RPM teams after implementing RPM, adjusting human resource allocation, and establishing new consultation workflows for handling alerts [20]. These findings support the broader concept that implementing RPM may require dedicated roles and adaptations to existing care processes.

In line with these insights, the proposed theoretical hybrid care pathway differs from the current care pathway by introducing a new stakeholder, the COPD coach, to support the integration of RPM into COPD care. While the name given to this new stakeholder is purely arbitrary, the role itself was proposed by workshop participants as a potential coordinating function for RPM-related activities. The COPD coach could be responsible for all RPM-related activities, including device installation, providing information on device use, following up on alerts, and discontinuing RPM. The COPD coach could perform these tasks in close collaboration with the treating pulmonologist and be responsible for reporting escalation outcomes to the treating GP.

Another stakeholder potentially involved in RPM activities is the informal caregiver, whose role in supporting patients throughout the disease trajectory has been repeatedly underscored by workshop participants but remains frequently underestimated [8]. Within the care pathway, the involvement of informal caregivers varies across phases of disease management but typically includes assisting with activities of daily living, medication management, emotional support, and coordination with HCPs [21]. Importantly, informal caregivers could also play an important role in the successful integration of RPM into the pathway. They may help patients operate monitoring devices, interpret and track generated health data, and liaise with the COPD care team (eg, the COPD coach) when alerts or issues arise. Empirical studies suggest that patients with engaged informal caregivers demonstrate improved symptom management, higher adherence to prescribed therapies, and more effective participation in self-management activities [22,23]. Consequently, informal caregivers act as an essential link between the patient, the RPM system, and the multidisciplinary care team, ensuring continuity, accuracy, and responsiveness throughout the proposed hybrid COPD care pathway.

While the COPD coach and informal caregiver play important roles in the proposed theoretical hybrid COPD care pathway, other stakeholders may also play significant roles. During the RPM follow-up period, initial triage and coordination are handled by the COPD coach, but the treating pulmonologist retains overall responsibility for the patient’s clinical management when RPM alerts are generated. In some cases, the GP may adjust medical treatment during a scheduled visit based on RPM data trends or clinician-initiated recommendations. When an informal caregiver is absent, home care nurses may support the patient in operating RPM devices or help interpret monitoring data. They may serve as the interface between the patient, caregiver, and physician, ensuring device use and reporting. Thus, RPM becomes embedded within a multistakeholder care model in which multiple stakeholders jointly contribute to surveillance, triage, and therapeutic decision-making [17].

Although this study did not focus on a specific RPM strategy, implementing RPM requires several considerations. First, a reliable device must be used to collect patient data, which may take the form of a dedicated physical device or an application installed on the patient’s smartphone. Second, data must be transmitted and stored automatically and securely, ensuring accessibility for authorized health care professionals. Where appropriate, algorithms for data processing and alert generation should be used to facilitate timely clinical decision-making. Third, the collected information should be presented in a meaningful and actionable manner for clinicians, ideally through integration with the electronic health record or other existing clinical systems [18]. Finally, patients must have sufficient digital literacy to participate effectively in an RPM program. Further research is needed to determine the level of digital literacy required for successful engagement [24].

In addition to RPM strategies, mobile health solutions can play a meaningful role in addressing educational needs. Mobile apps can provide structured, accessible, and on-demand educational content that reinforces disease knowledge, enhances understanding of treatment plans, and supports appropriate responses to changes in health status, potentially leading to reduced hospitalizations and improved quality of life [25,26]. Such digital tools may therefore complement traditional education, help overcome information loss after hospitalization, and strengthen patients’ self-management capacities [27].

### Future Perspectives

Future research should address several aspects related to the practical implementation of this theoretical framework. While previous research has demonstrated the feasibility and potential value of RPM using wearable devices in other care settings, further research is necessary to determine its impact on clinically relevant patient outcomes [28]. In addition, the influence of digital literacy and socioeconomic status on patients’ ability to participate in RPM programs should be further explored, as these factors have previously been identified as important considerations and potential barriers to the implementation of digital health technologies in clinical care [29]. Cognitive burden should also be further investigated, particularly in relation to patients’ ability and willingness to engage with RPM programs. Likewise, the influence of language proficiency and the availability and role of caregiver support should be explored, particularly among patients who may require additional support to engage with and use digital technologies.

Beyond the monitoring technology itself, future studies should further define strategies for alert management, as these are likely to influence the workload of the HCPs involved. The COPD coach role also requires further specification, including qualifications, training, workload, and patient-to-coach ratios, as well as clear procedures for alert escalation and clinical decision-making. In addition, practical and organizational considerations, including after-hours coverage, alert triage, reimbursement, and medicolegal responsibilities, need to be addressed before the role can be implemented in routine COPD care. Furthermore, the integration and communication of RPM data within existing health care networks, particularly between hospital-based and community-based care providers, should be evaluated in the context of local care structures. Finally, financial, organizational, and legal considerations require further investigation at the institutional and national levels. As these aspects are highly context-specific, they were beyond the scope of the current study.

### Study Limitations

This study has several limitations. First, the sample size was relatively small, and all participants were recruited from a limited geographical region in Belgium, which may limit the transferability of the findings to other settings. Second, patients who participated in the interviews had very low daily smartphone use (1 out of 5), potentially negatively influencing their opinions on the added value of RPM. As the digital literacy of the patient representatives in the workshops is unknown, there might be bias regarding the use of this technology in this patient population. Third, the use of convenience sampling of HCPs may have introduced selection bias. Fourth, only 1 informal caregiver (who also served as a patient representative) was included in the workshops. Further research is necessary on how informal caregivers can contribute to hybrid care for patients with COPD. Fifth, RPM was introduced as a wearable device, such as a smartwatch, during the workshops, which may have influenced participant views. Despite these limitations, this study offers valuable, in-depth insights into the perspectives of key stakeholders, providing a meaningful foundation for future research on the development of hybrid care pathways.

### Conclusion

This user-centered design study developed a theoretical hybrid COPD care pathway through interviews and workshops with stakeholders involved in COPD care. To facilitate the integration of RPM, stakeholders identified the need for a new role, namely, the COPD coach. This individual could be responsible for all RPM-related activities, including device installation, patient instruction, monitoring and triaging alerts, and managing the discontinuation of RPM services. A dedicated RPM role may support the coordination and integration of RPM into routine COPD care, as stakeholders highlighted that a digital tool alone may not be sufficient to achieve this. As the proposed pathway is purely a conceptual model, further research is necessary to evaluate long-term adherence in real-world clinical settings before implementation.

## Appendix

Multimedia Appendix 1 COREQ checklist.

Multimedia Appendix 2 Interview guide: patients.

Multimedia Appendix 3 Interview guide: physicians.

Multimedia Appendix 4 Interview guide: occupational therapists and physiotherapists.

Multimedia Appendix 5 Interview guide: chronic obstructive pulmonary disease (COPD) nurses.

Multimedia Appendix 6 Interview guide: remote patient monitoring (RPM) nurses.

Multimedia Appendix 7 Workshop guides for healthcare practitioners and patient representatives.

Multimedia Appendix 8 Infographic illustrating the proposed hybrid chronic obstructive pulmonary disease (COPD) care pathway. Changes from the current care pathway are marked with an asterisk (*).

## Abbreviations

ADL: activities of daily living
AECOPD: acute exacerbation of COPD
COPD: chronic obstructive pulmonary disease
COREQ: Consolidated Criteria for Reporting Qualitative Research
ER: emergency room
GP: general practitioner
HCP: health care professional
RPM: remote patient monitoring

## Data Availability

The data that support the findings of this study are available from the corresponding author upon reasonable request.
