# Supplementary material for: Constructing a Theoretical Hybrid Care Pathway From Stakeholder Perspectives: User-Centered Design Study
Source: JMIR Hum Factors. 2026 Oct 30;13:e93735. doi: 10.2196/93735 (PMC13768819; doi:10.2196/93735)
Supplement: Supplementary file 2 [file humanfactors_v13i1e93735_app8.pdf]

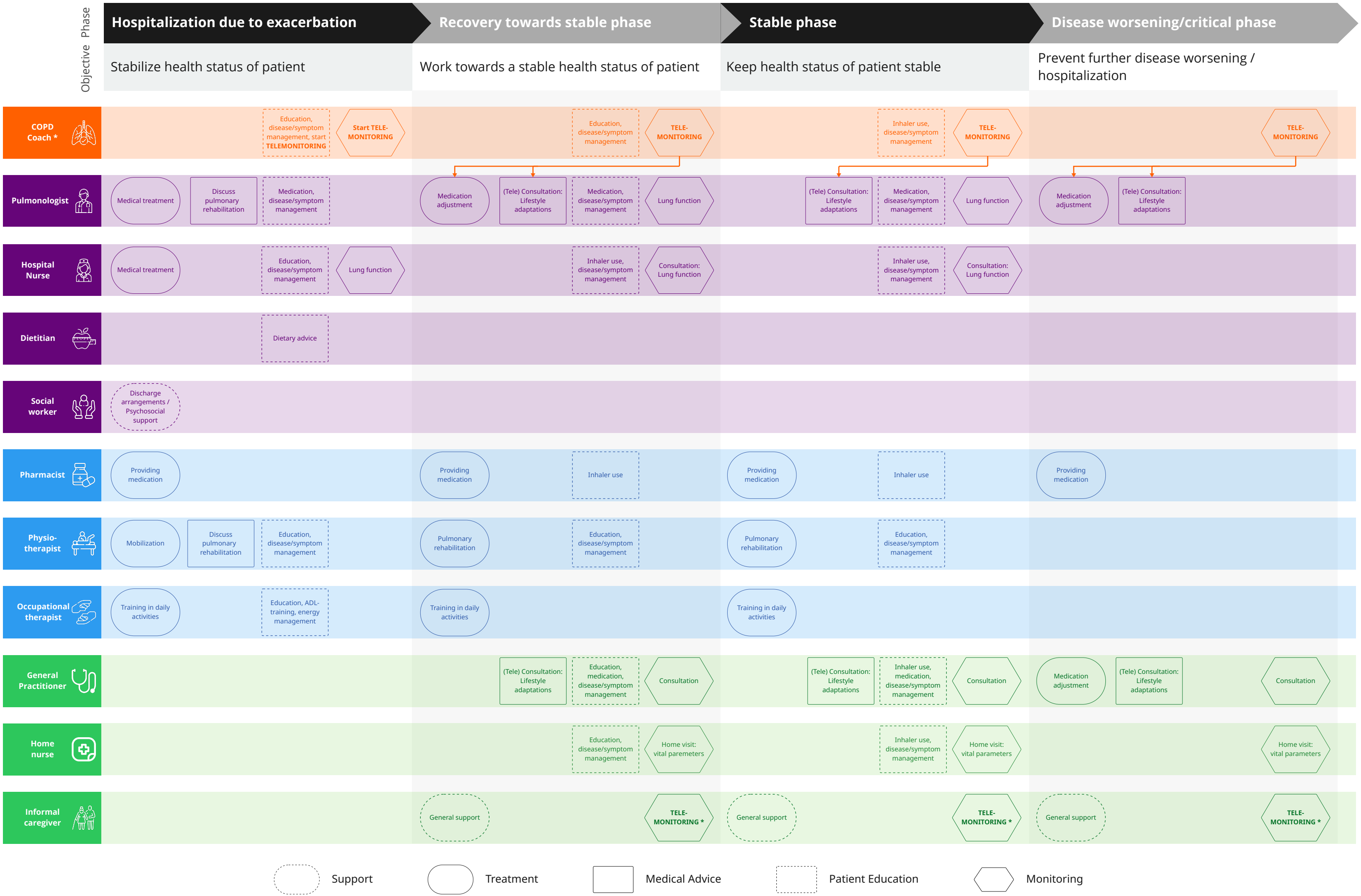

Phase
Objective
Hospitalization due to exacerbation
Recovery towards stable phase
Stable phase
Disease worsening/critical phase
Stabilize health status of patient
Work towards a stable health status of patient
Keep health status of patient stable
Prevent further disease worsening / hospitalization
COPD Coach *
Education, disease/symptom management, start TELEMONITORING
Start TELE-MONITORING
Education, disease/symptom management
TELE-MONITORING
Inhaler use, disease/symptom management
TELE-MONITORING
TELE-MONITORING
Pulmonologist
Medical treatment
Discuss pulmonary rehabilitation
Medication, disease/symptom management
Medication adjustment
(Tele) Consultation: Lifestyle adaptations
Medication, disease/symptom management
Lung function
(Tele) Consultation: Lifestyle adaptations
Medication, disease/symptom management
Lung function
Medication adjustment
(Tele) Consultation: Lifestyle adaptations
Hospital Nurse
Medical treatment
Education, disease/symptom management
Lung function
Inhaler use, disease/symptom management
Consultation: Lung function
Inhaler use, disease/symptom management
Consultation: Lung function
Dietitian
Dietary advice
Social worker
Discharge arrangements / Psychosocial support
Pharmacist
Providing medication
Providing medication
Inhaler use
Providing medication
Inhaler use
Providing medication
Physio-therapist
Mobilization
Discuss pulmonary rehabilitation
Education, disease/symptom management
Pulmonary rehabilitation
Education, disease/symptom management
Pulmonary rehabilitation
Education, disease/symptom management
Occupational therapist
Training in daily activities
Education, ADL-training, energy management
Training in daily activities
Training in daily activities
General Practitioner
(Tele) Consultation: Lifestyle adaptations
Education, medication, disease/symptom management
Consultation
(Tele) Consultation: Lifestyle adaptations
Inhaler use, medication, disease/symptom management
Consultation
Medication adjustment
(Tele) Consultation: Lifestyle adaptations
Consultation
Home nurse
Education, disease/symptom management
Home visit: vital paremeters
Inhaler use, disease/symptom management
Home visit: vital parameters
Home visit: vital parameters
Informal caregiver
General support
TELE-MONITORING *
General support
TELE-MONITORING *
General support
TELE-MONITORING *
Support
Treatment
Medical Advice
Patient Education
Monitoring
