# Supplementary material for: Constructing a Theoretical Hybrid Care Pathway From Stakeholder Perspectives: User-Centered Design Study
Source: JMIR Hum Factors. 2026 Oct 30;13:e93735. doi: 10.2196/93735 (PMC13768819; doi:10.2196/93735)
Supplement: Supplementary file 3 [file humanfactors_v13i1e93735_app2.docx]

RM@H-QS

*Interviews and workshops to improve patient care*

**Interview guide - patient**

Version number: 1.0

Version date: March 27, 2025

1. **Not recorded part**

*1.1 Key considerations for the researchers*

− Ask one question at a time (no double questions at once)
− Ask sufficient follow-up questions: ask for concrete examples, situations, both positive and negative aspects, …
− Regularly summarize, paraphrase and check (e.g. “Have I understood correctly that…”)

*1.2 Start:*

Welcome, thank you for being willing to participate in this research. Before we begin, I will ask you to sign the informed consent form, if you agree. If you wish, we can go through it together once more? Do you have any questions? Please feel free to ask, and then I can give you more information about the interview and the research. If everything is clear, we will begin the interview.

*1.3 Instructions prior to the interview:*

Today I would like to talk about the care pathway of COPD patients, what this looks like, who is involved and at which moments. This conversation is therefore about your experiences with the care pathway for COPD, from diagnosis to follow-up. We want to understand what goes well and where there are bottlenecks, so that we can think about improvements.

The conversation will be recorded and I will ask open questions. Please know that there are no right or wrong answers. Do you have any questions?

I will now start the recording and begin the conversation.

**2. START RECORDING**

*2.1 Introduction:*

Thank you for participating in our research. Before we start the open questions, I will ask a few short questions to get the conversation going.

1. What is your age and gender?
2. Do you remember when you were diagnosed with COPD?
3. How would you describe your digital and technological skills?

On a scale of never – rarely – sometimes – often – daily:

1. I use the internet
2. I use a computer and/or laptop
3. I use a smartphone and/or tablet
4. I use a wearable (fitness tracker, smartwatch or other)

4. Do you have experience with remote monitoring, such as digital tools or apps for health monitoring?

*1. Treatment after discharge from the hospital*

1. Can you describe what your treatment looked like after you were discharged from the hospital following a COPD exacerbation?
2. When were you discharged from the hospital?
3. How many days did you stay in the hospital?
4. On a scale of 1 to 10, how well did you feel prepared to go home?
5. At discharge, did you still have any symptoms that concerned you?

*2. Follow-up and further care*

6. What does the medical follow-up look like after discharge?
   − When and how often do you have standard consultations?
7. Have you been readmitted to the hospital after discharge?
   − How much time was there between two readmissions?
   − Have you noticed a pattern in your readmissions? (For example, seasonal, after exertion, after an infection, etc.)
8. Did you undergo rehabilitation after discharge? If yes, how did you experience this?
9. Which other healthcare professionals have you been in contact with?
10. Were there other aspects that were important in your care after discharge, such as medication use or home care?

*3. Experienced bottlenecks and areas for improvement*

11. Where and when did you feel that things went less well in your care? Have you experienced problems or are there things that could be improved?
    − At discharge from the hospital?
    − During medical follow-up?
    − During a readmission?
    − In communication between different healthcare professionals?

*4. Disease progression and symptoms*

12. How did you experience it when your health deteriorated?
    − Were there specific symptoms that you noticed before you deteriorated? (For example, a change in well-being, shortness of breath, fatigue, etc.)
13. Did you know who to contact if you felt worse?
    − Did you first contact your general practitioner?
    − Did you go directly to the emergency department?
    − Did other healthcare professionals play a role, such as a home nurse?
14. Has your medical follow-up changed after a readmission?
    − Did you have to come for check-ups more often or were you given appointments with new healthcare professionals?
    − Was this an improvement in your opinion?

*5. Support from informal caregivers*

15. Do you receive help from an informal caregiver/someone who takes care of you in your home environment?
    − What is their role in your care?
    − What does your informal caregiver/this person help you with?

*6. Improvements in COPD care*

16. If you could change something about COPD care, what would that be?

*3. STOP RECORDING*

Thank you again for your participation!
