# Supplementary material for: Constructing a Theoretical Hybrid Care Pathway From Stakeholder Perspectives: User-Centered Design Study
Source: JMIR Hum Factors. 2026 Oct 30;13:e93735. doi: 10.2196/93735 (PMC13768819; doi:10.2196/93735)
Supplement: Supplementary file 4 [file humanfactors_v13i1e93735_app3.docx]

RM@H-QS

*Interview and workshops to improve patient care*

**Interview guide - physician**

Version number: 1.0

Version date: March 27, 2025

1. **Not recorded part**

*1.1 Key considerations for the researchers*

− Ask one question at a time (no double questions at once)
− Ask sufficient follow-up questions: ask for concrete examples, situations, both positive and negative aspects, …
− Regularly summarize, paraphrase and check (e.g. “Have I understood correctly that…”)

*1.2 Start:*

Welcome, and thank you for your willingness to participate in this research. Before we begin, I will ask you to sign the informed consent form, if you agree. If you wish, we can go through it together once more? Do you have any questions? Please feel free to ask, and then I can give you more information about the interview and the research. If everything is clear, we will begin the interview.

*1.3 Instructions prior to the interview:*

Today I would like to talk about the care pathway of COPD patients, what this looks like, who is involved and at which moments. This conversation is therefore about your experiences with the care pathway for COPD, from diagnosis to follow-up. We want to understand what goes well and where there are bottlenecks, so that we can think about improvements.

The conversation will be recorded and I will ask open questions. Please know that there are no right or wrong answers. Do you have any questions?

I will now start the recording and begin the conversation.

2. **START RECORDING**

*2.1 Introduction:*

Thank you for participating in our research. Before we start with the open questions, I will ask a few short questions to get into the conversation.

1. What is your age and gender?
2. What is your position and specialization within COPD care?
3. How long have you been working with COPD patients?
4. How would you describe your digital and technological skills?
   1. On a scale of never – rarely – sometimes – often – daily:
      − I use the internet
      − I use a computer and/or laptop
      − I use a smartphone and/or tablet
      − I use a wearable (fitness tracker, smartwatch or other)
5. Do you have experience with remote monitoring for COPD patients?
   1. If yes, which tools or technologies have you used and in what context?

Now we will continue with questions that you may need a little more time to think about. These are also related to this diagram (infographic) that we have developed as a guide.

*1. Treatment after discharge from the hospital*

1. What does the standard treatment look like for a COPD patient after discharge from the hospital?
   − Are there specific protocols or guidelines that are followed?
2. Which criteria determine when a patient is discharged from the hospital?

*2. Follow-up and further care*

3. How does the follow-up of COPD patients proceed after discharge?
   − When and how often are standard consultations scheduled?
4. Which factors determine whether a patient is readmitted?
   − Are there specific criteria or thresholds for readmission?
5. When is a patient referred for rehabilitation?
6. Which other healthcare professionals are involved in the follow-up of the patient?
7. Are there additional elements that play a role in aftercare, such as medication, home care or other interventions?

*3. Experienced bottlenecks and areas for improvement*

8. Where and when do you think things go wrong in the care of COPD patients?
   − Are there specific problems or areas for improvement that you have noticed?

*4. Disease progression and preventive measures*

9. Which patients are more susceptible to a deterioration of their disease?
   − Do you take this into account in the follow-up?
   − Is proactive action taken, for example, with additional check-ups or monitoring?
10. What is the policy when the health of a COPD patient deteriorates?
    − Which treatments and follow-up strategies are then implemented?
11. What recommendations do you give to patients when their disease deteriorates?
    − Is it advised to go to the general practitioner first?
    − Or is it recommended to go directly to the emergency department?
    − Do other healthcare profiles, such as a monitoring nurse, play a role in this?

*5. Readmission and monitoring*

12. Which measures are taken to prevent readmissions?
13. How often do disease deterioration and readmissions occur?
    − Do you have an estimate of how many patients experience this?
14. When could you (theoretically) detect changes in vital parameters in COPD patients?
15. Does the care pathway of a patient change after a COPD exacerbation?
    − Are there structural improvements in care after a readmission?

*6. Role of informal caregivers*

16. What is the role of informal caregivers in the care of COPD patients?
    − What do they help the patient with?
    − What do you as a healthcare professional need to better support informal caregivers?

*7. Improvements in COPD care*

17. What do you as a healthcare professional need to provide better care to COPD patients?

That was the last question from our side. Was everything clear? Do you have any questions or comments? You can also contact me later if something comes to mind. I will now stop the recording.

*3. STOP RECORDING*

Thank you again for your participation!
