# Supplementary material for: Constructing a Theoretical Hybrid Care Pathway From Stakeholder Perspectives: User-Centered Design Study
Source: JMIR Hum Factors. 2026 Oct 30;13:e93735. doi: 10.2196/93735 (PMC13768819; doi:10.2196/93735)
Supplement: Supplementary file 5 [file humanfactors_v13i1e93735_app4.docx]

RM@H-QS

*Interviews and workshops to improve patient care*

**Interview guide – Occupational therapist & physiotherapist**

Version number: 1.0

Version date: March 27, 2025

1. **Not recorded part**

*1.1 Key considerations for the researchers*

− Ask one question at a time (no double questions at once)
− Ask sufficient follow-up questions: ask for concrete examples, situations, both positive and negative aspects, …
− Regularly summarize, paraphrase and check (e.g. “Have I understood correctly that…”)

*1.2 Start:*

Welcome, thank you for being willing to participate in this research. Before we begin, I will ask you to sign the informed consent form, if you agree. If you wish, we can go through it together once more? Do you have any questions? Please feel free to ask, and then I can give you more information about the interview and the research. If everything is clear, we will begin the interview.

*1.3 Instructions prior to the interview:*

Today I would like to talk about the care pathway of COPD patients, what this looks like, who is involved and at which moments. This conversation is therefore about your experiences with the care pathway for COPD, from diagnosis to follow-up. We want to understand what goes well and where there are bottlenecks, so that we can think about improvements.

The conversation will be recorded and I will ask open questions. Please know that there are no right or wrong answers. Do you have any questions?

I will now start the recording and begin the conversation.

**2. START RECORDING**

*2.1 Introduction and background*

1. What is your age and gender?
2. What is your current position and specialization (background)?
3. Which patient populations do you mainly work with? (e.g. only COPD, or also asthma, other pulmonary diseases…)
4. How long have you been working with pulmonary (COPD) patients?
5. How would you describe your digital skills?

− On a scale of never – rarely – sometimes – often – daily:

- I use the internet
- I use a computer/laptop
- I use a smartphone/tablet
- I work with medical data platforms
- I receive data from wearables or home measurements (e.g. pulse oximeter, spirometer)

6. Do you follow up with the patients? If yes, from admission or already from diagnosis? In what way? How does this proceed?
7. Which patients do you follow up with (e.g. only more severe patients)?
8. What does your range of responsibilities include?
   − Mobilization
   − Information and education
   − Motivation/coaching for treatment adherence
   − Advice on lifestyle or inhalation technique
   − Guidance on exacerbation prevention
   − Other
9. Is there some form of ‘remote’ monitoring? How do you monitor patients in hospital (e.g. monitoring oxygen saturation during exercises)?
10. Are the data platforms you work with linked to the electronic patient record (EPR)?
    − Is there an automatic connection or does the information have to be transferred manually?
    − Does the treating physician (pulmonologist / general practitioner) have direct access to the (telemonitoring) data?
    − Is there contact with home nurses/physiotherapists/occupational therapists in primary care?
    − If it is not linked, is the data nevertheless entered into the EPR (in another way)?

*2.2. Role as physiotherapist / occupational therapist and patient guidance*

11. How often and in what way do you have contact with the patient? (by telephone, video call, in person)
12. How much time do you spend on average per patient?
13. How do you actively involve the patient in their own disease and self-management?
14. How does the patient view this, what is their experience with this (are they positive)?
15. What skills do you need to perform your role?

*2.3. Telemonitoring in practice*

16. What types of data do you monitor in COPD patients?
    − Examples: oxygen saturation, respiratory rate, symptoms, cough frequency, lung function (FEV1), activity, weight… → which EPR do you have? Are there separate tabs that you fill in?
17. Do you also assess whether the patient is ‘ready for discharge’? Do you have a say in whether or not the patient is discharged? If not, would you like to?

*2.4. Current barriers and prerequisites*

22. What bottlenecks do you experience when following up with COPD patients (via telemonitoring)?
    − Think of: false alarms, technical problems, limited treatment adherence, language barriers, limited digital skills…
23. To what extent are patients actively involved in their own data?
24. What would be the ideal workflow for you for COPD patients?

*2.5. Future perspective*

28. How do you see the future of COPD coaching and telemonitoring evolving, and what role do you see for the physiotherapist / occupational therapist in this?
    − If applicable: do you see a role for telemonitoring in the COPD population?
    − A greater role for AI in early detection?
    − More integration into primary care?
    − More emphasis on self-management by patients?
29. What would you personally need to be able to do your work better?

*2.6. Conclusion*

30. Was everything clear?
31. Do you have any suggestions or other things you would like to share?

*2.7. STOP RECORDING*

Thank you again for your participation!
