# Supplementary material for: Constructing a Theoretical Hybrid Care Pathway From Stakeholder Perspectives: User-Centered Design Study
Source: JMIR Hum Factors. 2026 Oct 30;13:e93735. doi: 10.2196/93735 (PMC13768819; doi:10.2196/93735)
Supplement: Supplementary file 7 [file humanfactors_v13i1e93735_app6.docx]

RM@H-QS

*Interviews and workshops to improve patient care*

**Interview guide – RPM nurse**

Version number: 1.0

Version date: March 27, 2025

1. **Not recorded part**

*1.1 Key considerations for the researchers*

− Ask one question at a time (no double questions at once)
− Ask sufficient follow-up questions: ask for concrete examples, situations, both positive and negative aspects, …
− Regularly summarize, paraphrase and check (e.g. “Have I understood correctly that…”)

*1.2 Start:*

Welcome, thank you for being willing to participate in this research. Before we begin, I will ask you to sign the informed consent form, if you agree. If you wish, we can go through it together once more? Do you have any questions? Please feel free to ask, and then I can give you more information about the interview and the research. If everything is clear, we will begin the interview.

*1.3 Instructions prior to the interview:*

Today I would like to talk about the care pathway of COPD patients, what this looks like, who is involved and at which moments. This conversation is therefore about your experiences with the care pathway for COPD, from diagnosis to follow-up. We want to understand what goes well and where there are bottlenecks, so that we can think about improvements.

The conversation will be recorded and I will ask open questions. Please know that there are no right or wrong answers. Do you have any questions?

I will now start the recording and begin the conversation.

**2. START RECORDING**

*2.1 Introduction:*

Thank you for participating in our research. Before we start the open questions, I will ask a few short questions to get the conversation going.

1. What is your age and gender?
2. What is your current function and specialization?
3. Which patient groups do you primarily work with? (e.g., patients with pacemakers, heart failure, etc.)
4. How long have you been working with remote patient monitoring?
5. How would you describe your digital and technological skills?

On a scale of never – rarely – sometimes – often – daily:

1. I use the internet
2. I use a computer and/or laptop
3. I use a smartphone and/or tablet
4. I work with medical data platforms
5. I receive data from wearables or implantables

6. Are the data platforms you work with connected to the electronic patient record (EPR)?
   − Is there an automatic connection or does the information have to be transferred manually?
   − Does the treating physician (cardiologist / general practitioner) have direct access to the telemonitoring data?

*2.2 Telemonitoring in practice*

7. What types of data do you monitor in your current patient population?
   − For example heart rhythm, activity, respiratory rate, oxygen saturation, weight, ...
8. In what way does the data come in?
   − Real-time, daily overview, with a delay, upon events/alerts?
9. How does the follow-up specifically take place?
   − Do you receive a notification when there are abnormalities?
   − Do you follow up according to a fixed schedule?
10. Is there a protocol that must be followed when an alert comes in?
    − What are the possible steps following an alert? (e.g. contact the patient, change medication, notify the physician, …)
    − Who takes these steps? Is this centralized or shared among healthcare professionals?
11. Is there a record of what is done in response to an alert?
    − Is this logged in the platform, in the EPR, or nowhere?
    − Is it evaluated whether the action has had an effect?

*2.3 Current barriers and prerequisites*

12. What bottlenecks do you experience in monitoring patients through telemonitoring?
    − Think of: frequent false alarms, insufficient staff, data volume, interpretation, technical failures, ...
13. Are there situations in which monitoring data is ignored or cannot be followed up?
14. What technological or organizational support is currently lacking?
15. To what extent are patients actively involved in their own data?
16. What would be the ideal workflow for you?

*2.4 Translation to other patient populations (such as COPD)*

17. Do you think a similar approach would be possible for COPD patients? Why (not)?
18. Which parameters do you think could be useful in this group?
19. What specific considerations should there be for COPD compared with cardiac patients?

*2.5 Future perspective*

20. How do you see telemonitoring evolving in the future?
    − A greater role for AI?
    − More self-management for patients?
    − Integration into primary care?
21. What would you personally need to be able to do your work better?

*2.6 Conclusion*

22. Was everything clear?
23. Do you have any suggestions or other things you would like to share?

*2.7 STOP RECORDING*

Thank you again for your participation!
