# Supplementary material for: Constructing a Theoretical Hybrid Care Pathway From Stakeholder Perspectives: User-Centered Design Study
Source: JMIR Hum Factors. 2026 Oct 30;13:e93735. doi: 10.2196/93735 (PMC13768819; doi:10.2196/93735)
Supplement: Supplementary file 8 [file humanfactors_v13i1e93735_app7.docx]

**Workshop RM@H-QS - HCPs**

**Planning**

1. **Tour-de-table & explanation of project goals**
   - In an “ice breaking” way: Who are you, what is your expertise? What was your most enjoyable moment in the hospital? What is your favorite data tracking device?
2. **Explanation of problem statement & RPM tool**
3. **Presentation of and feedback on initial infographics based on the interviews we did before**
   - Zoom in on a common problem mentioned: the black box of what happens at home after discharge (i.e., hospitalization of COPD-exacerbation). “From your experience, what are the main challenges in this phase of care?”
     - Project goal: early detection of complications
     - What could be the cause of this problem (i.e., current inability to detect complications early)?
       - E.g., education / recognition /detection of complications is limited or too late
       - Health literacy of the patient / family
       - Lower socio-economic status / migrants
   - Based on which criteria are patients discharged?
     - Clinical criteria (e.g., vital parameters within certain range)
       - Vital parameters
       - No need for O2 anymore
     - Social criteria (e.g. readiness of home situation)
       - Self-management and motivation of the patient
       - Social wellbeing -> is this evaluated, by whom?
       - Who is available at home (e.g. informal caregiver / family?)
       - What is present at home (e.g. special bed, wheelchair)
   - Are roles and responsibilities of all stakeholders involved in the care path correctly presented, like?
     - Is the 1^st^ line (GP) involved? Does this meet requirements?
     - Is there a need for improvement in communication between primary and secondary care providers and if so, why / how?
     - Are there existing bottlenecks in the referral or follow-up process that impact patient outcomes?
     - Do patients contact the care team (too) often?
2. **Discuss RPM & roles and responsibilities:**
   - What do you expect from remote monitoring?
   - Show examples of remote monitoring
   - Do caregivers sense the urgence of a transformation to hybrid care?
     Starting point of RPM:
     - Post-hospital discharge
       - How can RPM complement or improve patient follow-up?
       - When should a patient be onboarded in the hybrid care path?
       - Who should onboard a patient in the hybrid care path?
       - Do the roles and responsibilities of the stakeholders change when introducing RPM?
         - When does your responsibility start / stop?
         - Whose responsibility is it to act on RPM alerts?
         - How do we ensure RPM data is meaningfully integrated into existing workflows without increasing workload for the HCP?
         - How and what information from RPM should be shared among stakeholders while respecting privacy regulations?
         - Who is the first contact person (in the current care path)?
         - Will the current contact person also be the contact person in the hybrid care path? (Or will this role be assigned to another HCP)?
       - Hurdles for patients to use RPM?
         - Note to workshop leader: If you ask this as an icebreaker, it could be good to log it and refer to it here. People with experience might see different challenges, compared to those without experience. You can also ask if they were told by others what their experiences were.
         - Bias: in possession of a smartphone/(fast)internet access/Wi-Fi at home
         - Digital literacy
         - Feeling comfortable with the use of apps and wearables (during daytime and night)
         - Compliance and adherence to wearing the wearable as continuous as possible?
         - Is it a burden to you to use RM (e.g., charging the device, receiving reminders and alerts,...)?
         - What are concerns when we would introduce RPM?
       - Added value/Advantages RPM?
   - Stopping RPM
     - What is the end-point of remained interest for the HCP or the patient?
     - What is the cut-off moment for patient remote monitoring?
       - How long does the high-risk complication period typically last?
       - Duration of RPM? Is there a specific point at which RPM should be/can be discontinued?
       - How long does a patient expect to be monitored?
       - How does a patient feel when remote monitoring is stopped? Anxious, happy?
   - Antigoal (make sure workshop does not go into detail of):
     - E.g. increase in workload for care/additional cost / new stakeholders (like 1^st^ line in the clinical pathway / unplanned discontinuation of RPM use by the patients (missing data)

**Workshop RM@H-QS – Patient representatives**

**Planning**

1. **Introduction and explanation of the project objectives**
1. **Tour-de-table & explanation of project goals**
   - Who are you?
   - Do you use a pedometer, smartwatch or health app? What do you like or dislike about it?
2. **Introduction to the project**
   - The project aims to develop new hybrid care pathways for people with chronic conditions, in which remote patient monitoring (RPM) also plays a role.
   - In this workshop, we would like to hear your views on how care currently works, what is going well, where improvements could be made, and what role you think RPM could play.
2. **Visualizing the current care pathway & feedback**

Show simple infographics of the pathway a patient typically follows before, during, and after a hospital admission.

1. Questions for participants

- Do you recognize yourself in this care pathway?
- What do you think is missing?

2. Focus on a recognizable problem

- What happens at home after you are discharged from hospital (e.g., after a COPD exacerbation)? For many people, this part of the care pathway feels like a “black box”.
- What challenges do you experience during this period?

3. Project objective: Early detection of problems after hospital discharge.

4. Follow-up questions:

- What do you think causes complications to sometimes be detected too late?
  - Is there enough information or knowledge?
  - Do you feel you know what to look out for and how to recognize an exacerbation?
  - Do language, education, or financial circumstances play a role?
- Were you given information about warning signs when you were discharged?
- Are the responsibilities of the different healthcare providers clear?
- Would you like your GP to be more involved?
- Do you think communication between your GP and specialist works well? Where are the difficulties?
- Are you aware of any problems with referrals or follow-up that make recovery more difficult?
- Do you tend to contact your healthcare team too often?

3. **Remote patient monitoring (RPM) & expectations**

1. What do you expect from RPM?

2. Show examples of RPM devices (e.g., on a slide or through a demonstration).

3. Do you see a need for digital applications in your follow-up care?

4. Questions for participants

- Have you ever used a device like this?
  - If yes: What was your experience?
  - If no: Would you be willing to try it? Why (not)?
- Possible barriers
  - Access: Do you have a smartphone or internet access at home?
  - Comfort: Would you feel comfortable using a wearable device or an app?
  - Burden: Does it seem like a lot of effort (e.g., charging, notifications, daily use)?
- Possible benefits
  - Would you feel safer after being discharged from hospital?
  - Do you think your doctor could respond more quickly if something went wrong?
- When should RPM start?
  - Immediately after discharge, or only later?
  - Who should explain how it works?
  - Who should respond to RPM alerts?
  - Would you like to be able to see your own data?
  - Would your current point of contact remain the same in the hybrid care pathway?
- Possible barriers for patients
  - Do you feel you have sufficient digital skills?
  - Would you be willing to wear a device day and night?
  - Would you find notifications or reminders annoying?
  - Do you have any concerns about privacy, costs, stress, etc.?
  - Would you be willing to pay for it?

4. **Stopping RPM**
   - How long would you expect to be monitored?
   - How would you feel if monitoring suddenly stopped? Relief? Uncertainty?
   - How much support do you need after discharge?
   - Who should decide when RPM stops — you, your doctor, or together?
   - **Note:** During this workshop, do not go into detail about organizational aspects such as additional workload for healthcare providers, costs, or the involvement of new stakeholders.

Is there anything we have not discussed that you think is important to mention?

Thank you for your participation. We will take your input into account. Later this week, we will map the other side of the picture and try to find a balanced approach that is valuable and workable for everyone.
